# Organizational health literacy in health care: Results from a participatory project in Germany

**DOI:** 10.1093/eurpub/ckac129.382

**Published:** 2022-10-25

**Authors:** K Rathmann, LD Salewski, LV Zelfl

**Affiliations:** 1 Department of Health Sciences, Fulda University of Applied Sciences, Fulda, Germany; 2 Public Health Centre Fulda, Fulda University of Applied Sciences, Fulda, Germany

## Abstract

**Background:**

Health care facilities (i.e., hospitals, care facilities and integration assistance) play an important role in providing health-related information and strengthening health literacy (HL) of patients/clients, staff and at the organizational level. The project “Development of Health Literacy in Health Care Facilities (EwiKo)’ aims at implementing tools to promote and strengthen organizational health literacy (OHL) in health care institutions in Germany. Objectives are 1) to assess needs for strengthening OHL in pilot facilities and 2) to examine factors that are beneficial to strengthen OHL in health care facilities.

**Methods:**

N = 6 pilot institutions (n = 2 hospitals, n = 2 care homes for elderly people, n = 2 facilities for people with disabilities) and their members of the “working groups on HL” filled in a self-assessment tool to assess the level of OHL, accompanied by focus group interviews (n = 6-9 persons per pilot facility). Regarding conducive and obstructive factors when implementing tools to strengthen OHL, focus group interviews (n = 6-9 persons per pilot facility) and semi-structured interviews (n = 1 coordinator per organization) were conducted.

**Results:**

Results of the self-assessment tool showed a need to strengthen OHL in all types of health care facilities, particularly in the fields of action ‘training of employees in HL tools’, “communication” and ‘participatory development and testing of documents, materials and services on HL’. Results of the focus group interviews showed a special need to strengthen OHL in the area ‘HL of employees’. Beneficial factors by implementing tools to strengthen OHL are, for instance a supporting management, resources, a participatory approach and trainings by the project team.

**Conclusions:**

Due to the ongoing corona pandemic and accompanying challenges (e.g., personnel resources), it is comprehensible that health care facilities emphasize a need for strengthening HL of employees.

